# *Pneumocystis* Pneumonia in Solid-Organ Transplant Recipients

**DOI:** 10.3390/jof1030293

**Published:** 2015-09-28

**Authors:** Xavier Iriart, Marine Le Bouar, Nassim Kamar, Antoine Berry

**Affiliations:** 1 Department of Parasitology-Mycology, Centre Hospitalier Universitaire de Toulouse, Hôpital Purpan, Institut Fédératif de biologie (IFB), 330 avenue de Grande Bretagne, TSA 40031, Toulouse 31059, France; E-Mail: lebouarmarine@gmail.com; 2 INSERM U1043, Toulouse F-31300, France; 3 CNRS UMR5282, Toulouse F-31300, France; 4 Université de Toulouse, UPS, Centre de Physiopathiologie de Toulouse Purpan (CPTP), Toulouse F-31300, France; 5 Department of Nephrology and Organ Transplantation, CHU Rangueil, TSA 50032, Toulouse 31059, France; E-Mail: kamar.n@chu-toulouse.fr

**Keywords:** *Pneumocystis jirovecii*, *Pneumocystis* pneumonia, transplantation, solid-organ transplant recipients

## Abstract

*Pneumocystis* pneumonia (PCP) is well known and described in AIDS patients. Due to the increasing use of cytotoxic and immunosuppressive therapies, the incidence of this infection has dramatically increased in the last years in patients with other predisposing immunodeficiencies and remains an important cause of morbidity and mortality in solid-organ transplant (SOT) recipients. PCP in HIV-negative patients, such as SOT patients, harbors some specificity compared to AIDS patients, which could change the medical management of these patients. This article summarizes the current knowledge on the epidemiology, risk factors, clinical manifestations, diagnoses, prevention, and treatment of *Pneumocystis* pneumonia in solid-organ transplant recipients, with a particular focus on the changes caused by the use of post-transplantation prophylaxis.

## 1. Introduction

*Pneumocystis jirovecii* is a ubiquitous and opportunistic fungus that is localized in the alveoli of human lungs and causes pneumonia. *Pneumocystis* pneumonia (PCP) remains a frequent cause of infection among immunocompromised patients [1,2].

*P. jirovecii* specifically infects humans, and PCP is an anthroponosis where humans are the only reservoir [3]. Rodent models and human observations have demonstrated inter-individual airborne transmission of PCP [3,4]. Recent data suggest that the cystic forms (but not the trophic forms) are responsible for transmission [5,6]. Transmission can occur from patients with PCP but also from asymptomatic hosts that can be transiently infected with *Pneumocystis* (colonization) [7,8,9]. Several data suggest that PCP is not a result of reactivation of a quiescent stage, such as cerebral toxoplasmosis, but rather is caused by direct infections or reinfections [10].

PCP was principally observed in HIV-positive patients in the 1990s [11] and is now well known and described in this population. Nevertheless, its incidence has dramatically increased over the last 15 years in patients with other predisposing immunodeficiencies [12,13,14,15], particularly transplant recipients [2,16,17,18]. Because of this emergence, it is necessary to know the specificity of PCP in other immunocompromised HIV-negative patients. Indeed, comparative studies on *P. jirovecii-*infected patients with AIDS, or with other immunodeficient conditions including transplantation, have shown substantial specificities in the patients’ demographics, and their biological and clinical presentations, outcomes, and mortality rates [19,20]. The objective of this review is not to develop official recommendations or to replace expert consensus, but to summarize the current knowledge on the epidemiology, risk factors, clinical manifestations, diagnoses, prevention, and treatment of *Pneumocystis* pneumonia in solid-organ transplant (SOT) recipients, taking into account the specificities of the medical management of these patients.

## 2. Epidemiology of SOT Recipients

### 2.1. Incidence

Since the 2000s, the ratio of *Pneumocystis* pneumonia in immunocompromised HIV-negative patients has increased rapidly [12,13,14,21]. Today, these patients represent the majority of PCP cases and underlying conditions include hematological malignancies, tumoral pathologies, hematopoietic stem cell transplantation, autoimmune or chronic inflammatory diseases, and solid-organ transplantation [22].

In more recent studies, SOT recipients represent ~30% of HIV-negative immunocompromised patients. Cases are mainly in kidney-transplant recipients because this is the main type of transplantation [22]. The most recent studies report that *Pneumocystis* infects 0.3%–2.6% of SOT recipients [23,24,25,26,27] whereas historic attack rates of PCP after solid-organ transplantation, defined as the biostatistical measure of frequency of morbidity in an at risk population, were ~5%–15% [16,28,29]. This decrease over time is probably because of the use of primary and prolonged prophylaxis with trimethoprim-sulfamethoxazole (TMP-SMX) today, as well as the use of modern immunosuppressive regimens [23].

The rate of PCP infection depends on the type of transplantation and is greater in heart-, lung-, and combined heart–lung transplantation than in kidney- or liver-transplant recipients, regardless of whether they do or do not receive prophylaxis (Table 1). This is probably because thoracic transplants recipients receive more intensive immunosuppressive therapies [30].

**Table 1:** Attack rate of *Pneumocystis* pneumonia (PCP) in transplant recipients. Adapted from Rodriguez *et al.* [31].

Organ Transplanted	Patients not Receiving Prophylaxis		Patients Receiving Prophylaxis *	
	Attack Rate (%)	Reference	Attack Rate (%)	Reference
Kidney	0.6–14	[30,32,33,34]	0.4–2.2 ^a^	[16,24,25,27]
Liver	3–11	[35,36,37]	1.1–3.7	[16,25,27,38]
Heart	2–41	[30,39,40,41]	2–5.1	[16,27]
Heart–lung/lung	6.5–43	[39,42]	5–5.8	[16,25]

* Early post-transplantation prophylaxis given for 6 months to 1 year. ^a^ Data from the study from Radisic *et al.* [43] has not been included (Attack rate: 5.4%) because of a preponderance of patients that had failed primary TMP-SMX prophylaxis.

In studies in which early post-transplantation prophylaxis against PCP was used, the attack rates of infection were drastically decreased for all types of transplantation (Table 1). The reported incidence rates of infection are 22 per 1000 patient-years for lung-transplant recipients, 0.14–7.3 per 1000 patient-years for heart-transplant recipients, 0.4–2.7 per 1000 patient-years for kidney-transplant recipients, and 0.2–10 per 1000 patient-years for liver-transplant recipients [16,27,44].

### 2.2. The High-Risk Period for PCP Development

The period of highest risk when PCP develops changes according to the use of early prophylaxis with TMP-SMX. Before the generalized use of prophylaxis, the highest risk period for developing PCP was the first six months after transplantation [33,45,46,47,48,49,50], particularly between the second and sixth month post-transplantation, and during periods of intensified immunosuppression [26].

**Figure 1:**
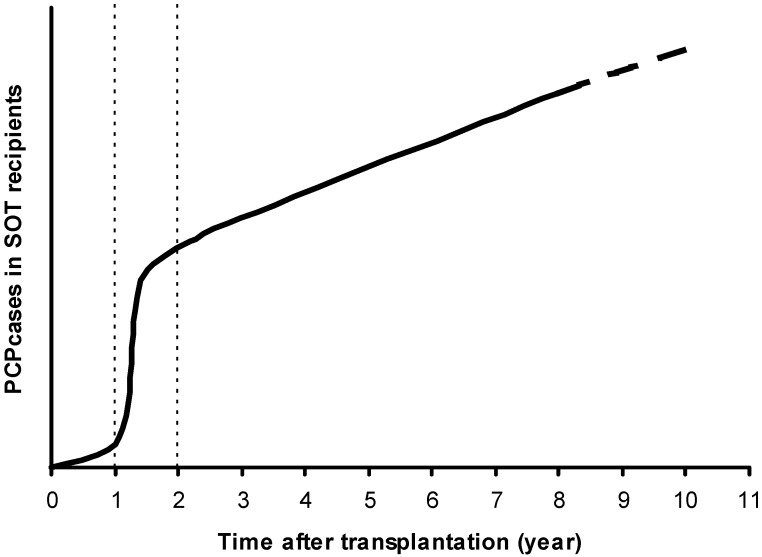
Risk of *Pneumocystis* pneumonia (PCP) at post-transplantation over time in patients that benefited from early prophylaxis with trimethoprim-sulfamethoxazole (TMP-SMX). Adapted from Iriart *et al.* [27]. The two dotted lines (1 and 2 years) delimit three different periods in the occurrence of the disease after the transplantation.

Since the establishment of routine TMP-SMX (or an alternative agent such as dapsone or atovaquone) prophylaxis, PCP within the first year post-transplant, has been virtually eliminated [13,51,52,53] and, today, this infection seems to occur mainly after the first year (Figure 1). Currently, the second year post-transplantation has a greater risk period of PCP with a high incidence in patients who have benefited from TMP-SMX prophylaxis within the first year [24,27]. However, the global incidence of SOT patients that develop PCP during the second year post-transplant is relatively low (<0.6%) [27].

Early prophylaxis seems to greatly reduce the risk of PCP during the first year but not within the second year. However, this is not a rebound effect, as second-year post-prophylaxis rates are comparable to second-year rates in centers that do not prescribe post-transplantation prophylaxis [26]. After the second year, the risk is lower and then remains so, but some risk persists for long after transplantation [24,27], possibly for the patient’s lifetime.

### 2.3. Seasonal Incidence

General data on a possible seasonal incidence of PCP are relatively discordant according to the studies. In two studies from the UK and Spain, a positive correlation was found between PCP incidence and colder months [54,55,56] but a higher incidence of PCP was found to be associated with the summer period in a recent study from Germany [57]. No seasonal variation of mortality due to PCP in HIV-infected individuals was observed in the Miller *et al*. study from the UK [58]. These discordances can be explained by the existence of other factors linked to climate, as, for example, human behavior, that outweigh the influence of single climatic factors. A systematic review of 10 studies reporting outbreaks of PCP in kidney transplant recipients shows no determining influence of climatic conditions [59]. The timing of peak incidence occurred during winter in four studies, during spring in one and in summer or early fall in five out of 10 studies, and no association between the time of diagnosis of the index case and any month or season [59].

### 2.4. Nosocomial Outbreaks

In transplant units, cases of PCP are often isolated but nosocomial outbreaks have been described, particularly for kidney-transplant recipients [48,60,61,62,63,64,65]. This is probably related to the high number of immunocompromised kidney SOT patients worldwide, plus their need for regular follow-up visits within hospital settings, involving potential inter-individual transmissions between receptive patients, colonized medical workers or colonized patients [66]. Nevertheless, a common source of exposure linked within transplant units cannot be excluded, as suggested by studies that detected *Pneumocystis* DNA in air samples from hospital-patient care rooms [67,68].

## 3. Risk Factors

Some risk factors for PCP are common, with these classically observed in all types of patients whereas other risk factors are specific to SOT recipients.

### 3.1. Immunosuppressive Regimens

The main risk factors for contracting PCP in SOT recipients are associated with immunosuppressant therapies. As observed during acute rejection episodes, the relative risk of PCP increases with the number of rejection treatment [26,43,50]; however, the contribution of individual immunosuppressants remains uncertain [69]. In several studies, the occurrence of PCP in SOT recipients has been directly linked to specific immunosuppressive regimens [33,70,71,72]. One of the more comprehensive studies on this topic was reported in the United States Renal Data System (USRDS) analyses, which included 142 patients with PCP and 32,615 controls [71]. Table 2 reports these results.

**Table 2:** Immunosuppressive regimens reported within the United States Renal Data System analyses. A total of 32,757 medicare primary adult recipients of a kidney transplant between 2000 and 2004. Adapted from Neff *et al.* [71].

N	PCP (%)	Controls (%)	*p*-Value; OR (95%CI)
	142	32,615	-
*Induction Therapy*			
None	33 (23.2)	5922 (18.2)	0.12
Thymoglobulin	29 (20.4)	7783 (23.9)	0.37
IL-2 receptor antibody	52 (36.6)	12,389 (38.0)	0.79
Alemtuzumab	2 (1.4)	476 (1.5)	0.96
*Discharge Immunosuppression*			
Tacrolimus	79 (55.6)	18,878 (57.9)	0.61
Cyclosporine (Neoral)	48 (33.8)	8755 (26.8)	0.07
Mycophenolate mofetil	96 (67.6)	25,415 (77.9)	**0.004**
Mycophenolate sodium	0 (0)	89 (0.3)	0.68
Azathioprine	7 (4.9)	900 (2.8)	0.12
Sirolimus	41 (28.9)	4857 (14.9)	**<0.001**
Steroids	124 (87.3)	28,916 (88.7)	0.61
*Discharge Immunosuppression by Combinations ^b^*			
Tacrolimus and mycophenolate mofetil	39 (27.5)	14,715 (45.1)	Reference 1.0
Tacrolimus and sirolimus	21 (14.8)	1861 (5.7)	**<0.001; 4.26 (2.50–7.25)**
Cyclosporine (Neoral) and mycophenolate mofetil	39 (27.5)	6762 (20.7)	**0.001; 2.18 (1.40–3.40)**
Sirolimus and mycophenolate mofetil	12 (8.5)	1544 (4.7)	**0.001; 2.90 (1.53–5.61)**
All other combinations	31 (21.8)	7733 (23.7)	0.09; 1.51(0.94–2.42)

^b^ *p*-value and OR are from unadjusted logistic regression. OR (95%CI): odds ratio (95% confidence interval). Bold indicates a *p*-value <0.05.

Nevertheless, data on the risk factors associated with immunosuppressive regimens are often contradictory due to the lack of homogeneity in drug dosages, the types of combinations used, the periods and sizes of the studies, and the use of PCP prophylaxis.

#### 3.1.1. Polyclonal/Monoclonal Antibodies

Induction therapies, particularly those that include polyclonal antibodies that deplete the lymphocyte pool, including CD4+ lymphocytes [73,74,75], are linked to a high risk of PCP during the first six months post-transplantation [76]. This risk of PCP is shared with populations with connective tissue diseases [15], who have also exposure to biological agents that may predispose to PCP infection, including TNF, CD20, or CD52 depleting therapies [77]. Nevertheless, no risk factors linked with induction therapies were identified in the USRDS analyses in SOT patients [71] (Table 2). Monoclonal antibodies, such as alemtuzumab or rituximab, used as induction therapies or to treat acute rejection in solid-organ transplantation, have been shown to confer a high risk of PCP in patients with hematological malignancies [77,78]. Few data are available concerning the risk of PCP associated with the use of IL2-receptor antagonists (IL2-RA). In Eitner *et al.*’s study, a lower rate of PCP infection was observed in patients treated with an IL2-RA [79]. To explain this finding, the authors hypothesized that either this drug had a direct action on *Pneumocystis* or IL-2RA acted indirectly by reducing the risk of rejection episodes and thereby reducing the need for immunosuppressive-associated treatments [79].

#### 3.1.2. Corticosteroids

Corticosteroids interfere with many pathways within the immune system [80], and they decrease the number and function of different cell populations, such as monocytes and macrophages [81,82], as well as peripheral blood CD4+ lymphocytes [83]. Corticosteroids have been clearly associated with a significant risk for PCP in non-HIV patients [84,85]. Although this corticosteroid-linked risk was not identified in the USRDS analyses [71] (Table 2), De Castro *et al.* showed that a longer duration of high-dose steroid therapy (>0.25 mg/kg per day) was associated with a higher risk of PCP in recipients of a kidney transplant (OR 1.6 per month, 95%CI: 1.04–2.93, *p* = 0.005) [86].

#### 3.1.3. Mycophenolate Acid

Mycophenolate acid (MPA) inhibits an enzyme implicated in purine synthesis, which is linked with the proliferation of B and T lymphocytes [87]. The first *in vitro* animal models indicated that MPA had an anti-*Pneumocystis* effect [88]. Furthermore, three clinical trials that evaluated MPA reported no cases of PCP in those that received MPA compared to 1.2%–2.4% of those that did not [89,90,91]. The USRDS data are consistent in showing that MPA was more frequently given to uninfected controls (Table 2) [71]. However, even though some authors have raised the question of the necessity of giving PCP prophylaxis when MPA is used [92], many cases of PCP have been reported in patients receiving MPA [23,71,93] and two recent retrospective case-control studies from Eitner *et al.* and Rostved *et al.* report an increased number of cases of PCP in MPA-treated patients. These data suggest that MPA alone or in combination did not confer any effective protection but, rather, was associated with an increased incidence of PCP [79,94].

#### 3.1.4. Calcineurin Inhibitors

Calcineurin inhibitors, including cyclosporine and tacrolimus, reduce the proliferation and differentiation of T lymphocytes by preventing interleukin-2 (IL-2) production. A first case series and experimental studies suggest that cyclosporine increases the risk of PCP in contrast to azathioprine or MPA [72,88,95]. For example, Hardy *et al.* observed an increase in the incidence of PCP from 3% to 9% when cyclosporine replaced azathioprine in kidney-transplant recipients at their institution [45]. Nevertheless, the retrospective study of Luft *et al.* showed a higher incidence of PCP among kidney-transplant recipients receiving a tacrolimus-based regimen compared to one based on cyclosporine [33].

More recently, the USRDS analyses did not identify tacrolimus as a risk factor for PCP (*p* = 0.61), whereas the use of cyclosporine was associated with a non-significant trend towards developing PCP (*p* = 0.07) [71] (Table 2). Conversely, in the case-control study of De Castro *et al.*, SOT recipients that contracted PCP were less likely to have received calcineurin inhibitors compared to those without PCP (OR 0.09, 95%CI 0–0.67, *p* = 0.004) [86]. These results were confirmed in a recent study by Iriart *et al.*, which also identified tacrolimus as a protective factor [27].

#### 3.1.5. mTOR Inhibitors

Sirolimus inhibits the response to interleukin-2, thus blocking the activation of T and B cells [77]. Initial studies on sirolimus also report high levels of PCP [70,96]. Two recent case-controls studies have confirmed an association between the use of sirolimus and the development of PCP: *i.e.*, De Castro *et al.* (OR 7.7, 95%CI 1.2–**∞**, *p* = 0.02) [86] and Neff *et al.* (*p* < 0.001) [71] (Table 2), as well as a study on pediatric recipients with an intestinal transplant [97].

#### 3.1.6. Combinations

In the USRDS review, maintenance immunosuppression regimens containing the combination of tacrolimus plus sirolimus, cyclosporin plus mycophenolate mofetil, and sirolimus plus mycophenolate mofetil were found to be significant risk factors for contracting a *Pneumocystis* infection [71] (Table 2).

### 3.2. Acute Rejection

The risk of PCP is also increased during phases when immunosuppression is enhanced, such as during an acute rejection. Five case-control studies identified a relationship between graft rejection and the risk of PCP [26,43,50,79,86] (Table 3). Even if the number of rejection episodes appeared to be a risk factor in Radisic *et al.*’s study [43], the number of treatments associated with acute-rejection episodes seemed to be the main parameter that accounted for this risk, irrespective of the drugs used.

**Table 3:** Identification of graft rejection as a risk factor for *Pneumocystis* pneumonia (PCP) in case-control studies.

Study	Prophylaxis	PCP Patients, *n*	Timing of Rejection (Post-Transplantation-Day)	Evaluated Criteria	Statistical Analysis	OR (95%CI)
Arend *et al.* 1996 [50]	No	15	1st episode: 20 2nd episode: 34 3rd episode: 57	No. of rejection treatment	Trend in relative risk (*p* = 0.002)	For 3 or more: 9.5 (1.6–56.4)
Radisic *et al.* 2003 [43]	1 year	17	1st episode: 8 2nd episode: 49 3rd episode: 62	Rejection episode	Univ (*p* = 0.02)	NA
				No. of rejection treatment	Trend in relative risk (*p* = 0.021)	For 3 or more: 6.3 (NA–NA)
				Type of rejection (steroid resistant)	Univ (*p* = 0.019)	4.3 (1.04–18.9)
De Castro *et al.* 2010 [86]	3 months	11	NA	Rejection episode	Univ (*p* = 0.002) Multiv (*p* = 0.017)	14.4 (2.1-inf) 8.7 (1.2-inf)
Eitner *et al.* 2011 [79]	No	60	NA	Biopsy-proven acute rejection episode	Univ (*p* = 0.0029)	NA
De Boer *et al.* 2011 [26]	No	50	1st episode: 16 Last episode: 48	Rejection treatment	Multiv (*p* = 0.002)	5.8 (1.9–17.5)
				No. of rejection treatment	Univ (*p* = 0.001)	For 3 or more: 12.9 (3.0–56.3)

NA: not available; OR (95%CI): odds ratio (95% confidence interval); Univ: univariate analysis; Multiv: multivariate analysis.

Because patients with a graft rejection receive a heavier burden of immunosuppression, they are more susceptible to PCP and other opportunistic infections. The use of polyclonal/monoclonal anti-lymphocyte antibodies in this context or patients who have a steroid-resistant allograft rejection seem to have additional risk factors for PCP (OR: 7.2 (95%CI: 1.3–49.3) and 4.3 (95%CI: 1.04–18.9), respectively) [43]. Because of the high number of kidney transplantations, these data mainly concern this population of patients but the risk of graft rejection has been also identified in adult liver-transplant recipients [38]. Except for two studies in which prophylaxis was either initiated for a short duration (three months) [86] or was associated with a high rate of failure for an undetermined reason [43], this risk was mainly identified in studies that included patients who had not received primary PCP prophylaxis (Table 3). However, in recent studies where patients benefited from six months of PCP prophylaxis, the risk factors associated with graft rejection were not confirmed [27,65]: perhaps graft rejection happened generally during the period covered by primary post-transplantation TMP-SMX prophylaxis.

### 3.3. Comorbidity Conditions and Co-Infections

Comorbidity conditions, such as underlying pulmonary diseases, transplant dysfunction [65], cancer, or transplant-donor factors [71], are more frequent in SOT recipient who develop PCP. Concomitant infections, such as tuberculosis, bacterial pneumonia, and hepatitis C infection, have also been associated with a risk of PCP [43], but cytomegalovirus (CMV) infection seems to be the co-infection that plays the most significant role in affects PCP SOT recipients.

Several case-control studies have identified CMV as a clear risk factor for PCP in SOT patients [26,43,50,65,71,98], regardless of whether these patients benefited from prophylaxis for PCP or not (Table 4). Except for the Arend *et al.*’s study [50], the CMV donor’s/recipient’s serostatus at transplantation was not significantly associated with PCP, even if the donor was CMV-positive and the recipient was CMV-negative [26,27,71].

The risk for PCP is mainly associated with patients who have had a CMV infection or CMV disease after transplantation [26,27,43,50,71,98]. Table 4 shows that 20%–53% of SOT recipients with PCP had a CMV infection compared to 0.6%–24% of those who did not develop PCP.

These CMV infections were mainly caused by CMV reactivation rather than a primary CMV infection [27]. However, it is unclear whether CMV infections simply reflect the degree of immunosuppression [16,33,45,47,48] or if CMV can directly increase the risk of PCP [50]. Nevertheless, it is noteworthy that cytomegalovirus has an immunosuppressive effect and causes marked impairment in cell-mediated immunity [99], which could reinforce the idea that CMV infection is a causal risk factor for PCP, independent of immunosuppression level [50].

**Table 4:** Identification of cytomegalovirus (CMV) as a risk factor for *Pneumocystis* pneumonia (PCP) in case-control studies.

Study	Prophylaxis	PCP Patients, *n*	Evaluated Criteria	CMV in PCP Cases, %	CMV in Controls, %	Statistical Analyses	OR (95%CI)
Arend *et al.* 1996 [50]	No	15	CMV infection	53.3%	18.8%	Univ (*p* < 0.05)	5.0 (1.6–15.8)
						Multiv (*p* < 0.05)	
Radisic *et al.* 2003 [43]	1 year	17	CMV infection	52.9%	23.5%	Univ (*p* = 0.036)	NA
Neff *et al.* 2009 [71]	NA	142	CMV disease	20.4%	8.8%	Univ (*p* < 0.001)	-
						Multiv: NS	
de Boer *et al.* 2011 [26]	No	50	CMV infection	NA	NA	Univ (*p* = 0.02)	2.7 (1.2–6.2)
						Multiv (*p* < 0.05)	3.0 (1.2–7.9)
Phipps *et al.* 2011 [65]	6 months	14	CMV disease	35.7%	0.6%	Univ (*p* < 0.001)	65.9 (7.9–550)
						Multiv (*p* < 0.001)	
Pliquett *et al.* 2012 [98]	No	29	CMV infection and disease	41.4%	3.4%	Univ (*p* ≤ 0.05)	NA
Rostved *et al.* 2013 [94]	No ^a^	16	CMV infection	31% ^b^ 29% ^c^	6% ^b^ 0% ^c^	Univ (*p* = 0.03) ^b^	NA
						Univ (*p* = 0.009) ^c^	
Iriart *et al.* 2015 [27]	6 months	33	CMV infection	51.5%	18.2%	Univ (*p* < 0.001)	5.2 (1.8–14.7)
						Multiv (*p* = 0.002)	

NA: not available; NS: not significant; OR (95%CI): odds ratio (95% confidence interval); Univ: univariate analysis; Multiv: multivariate analysis. ^a^ Initiated at the end of the outbreak; ^b^ kidney-transplant recipient; ^c^ liver-transplant recipient.

### 3.4. Blood Parameters

Some parameters that reflect the host-immune response are associated with a higher risk of PCP. The number and functional capacity of lymphocytes are especially important in a host’s defenses against *Pneumocystis* [100]. A decreased lymphocyte count, particularly of CD4+ T-cells, has been clearly linked to PCP in HIV-positive patients [101]. Even though blood CD4+ T-cell levels seem to less accurately reflect the risk of PCP in HIV-negative patients compared to alveolar CD4+ T lymphocyte counts [20], CD4+ T-cell lymphopenia has been also associated with PCP in HIV-negative patients [102], particularly in hematopoietic stem cell transplantation recipients [103], in solid-tumor patients receiving chemotherapy [84], in those with an autoimmune disease, and those with a hematological malignancy [104].

Recently, three studies have demonstrated that CD4+ T-cell lymphopenia or a lower total lymphocyte count are also risk factors for PCP in transplant recipients [27,29,86], although with some differences in threshold levels (750/µL and 500/µL in Iriart *et al.*’s and De Castro *et al.*’s studies, respectively [27,86]) and the decay kinetics of lymphocytes. Indeed, Struijk *et al.* reported that a significant decrease in lymphocytes occurred for about two years before PCP developed [29], whereas this decrease was only significant at ~50 days before PCP was evident in Iriart *et al.*’s study [27].

However, overall, these data suggest that lymphocyte counts may help guide an indication for chemoprophylaxis in these patients. Moreover, a lower total gamma globulin concentration in SOT patients with PCP was identified as risk factor in one case-control study [27], suggesting an alteration of the humoral immunity response, which plays a significant role against *Pneumocystis* [105]. In addition, prolonged neutropenia is a potential risk factor for PCP in transplant recipients, as observed in patients with a hematological malignancy [32].

### 3.5. Risk Period for PCP in SOT Recipients

As mentioned above, TMP-SMX prophylaxis influences when the highest risk of PCP occurs. Before the use of prophylaxis, this period was the first six months after transplantation [33,45,46,47,48,49,50], during the period of intensified immunosuppression [26]. Today, now that primary prophylaxis is usually used, PCP is rare within the first year [13,51,52,53], but the second year post-transplantation is now a new high-risk period for PCP [24,27]. After the second year, the risk is lower, but then persists at a relatively constant level, probably for the entire lifetime of the SOT recipient [24,27].

### 3.6. Nosocomial Risk

Failures of prophylaxis can also be linked to outbreaks of PCP [98,106], which are particularly frequent in renal-transplant units [60,61,62,63,106,107], although close contact with healthcare-associated clusters of PCP infection may also constitute a nosocomial risk factor for SOT recipients.

### 3.7. Characteristics of SOT Recipients

Age appears to be an independent risk factor for PCP, both in studies that have focused on HIV-positive or -negative patients with PCP, such as SOT recipients [26,27]. Risks seem to be increased for SOT recipients aged >55 years (OR 2.7, 95%CI 1.3–5.9) and the risk increases with age [26,27]. Female gender was associated with a higher incidence of PCP in one study [71]. The risk of PCP is higher in heart-, lung-, and combined heart–lung transplant recipients than in kidney- or liver-transplant recipients regardless of them receiving or not receiving prophylaxis (Table 1): this is probably due to the more intense immunosuppressive therapy given to the first patients [30].

The colonization of *Pneumocystis* also plays a role in the transmission of this fungus. This has been observed in healthy individuals but also in patients at risk for PCP. Although no study on SOT patients has demonstrated that colonization is an independent risk factor for PCP, several data suggest that colonization can precede infection [93].

### 3.8. Strategies to Target Patients at Risk for PCP

In two studies, the authors proposed strategies to target SOT recipients at high risk for PCP using risk factors commonly identified in this population [26,27]. The first study selected the most efficacious prophylactic strategies given within the first two years post-transplantation in patients who had not benefited from a prophylaxis [26]. The second study focused on patients with a high risk of PCP after the first year, and on SOT recipients who had benefited from initial TMP-SMX prophylaxis [27]. When age, the date of transplantation, any rejection episodes, lymphocyte counts, and detectable CMV viremia were considered in these scenarios, the prescription for PCP chemoprophylaxis according these criteria would result in a lower incidence of PCP and greater avoidance of TMP-SMX toxicity [108].

## 4. Clinical Manifestations and Outcomes

### 4.1. Clinical Manifestations

Whereas HIV-positive patients present with a subacute course and progressive deterioration of respiratory status (symptom duration: 25–28 days), symptomatic progression of PCP is, in general, more acute in HIV-negative patients (symptom duration: 5–6 days) [109,110]. In HIV-negative patients, common PCP symptoms, such as fever [111], are sometimes lacking because immunosuppressive agents have suppressed the clinical findings [76]. In the setting of transplantation, symptoms often develop over the course of a few days, although evolution over 1–2 weeks can also occur [111].

Pneumonia is the primary manifestation of a *P. jirovecii* infection. Common, but non-specific associated symptoms are fever, dyspnea, and a dry cough. Respiratory involvement is usually more severe in HIV-negative patients, with low arterial-oxygen tension and more frequent acute respiratory failure [109,110,112]. Table 5 reports the frequency of clinical presentations of PCP among kidney-transplant recipients in recent studies. Examinations of the lungs are often normal, even if there is hypoxemic presentation, but discrete crackles may be present [113]. A pneumothorax, often associated with acute dyspnea and pleuritic chest pain, may be observed, but is rare [114].

**Table 5:** Clinical presentations, radiological findings, and outcomes from *Pneumocystis* pneumonia (PCP) among kidney-transplant recipients in studies published after 2010.

Clinical Criteria	Reference	Frequency (%)
Clinical Presentation		
Cough	[29,86]	78%–100%
Fever	[29,86]	67%–100%
Dyspnea	[29,86]	73%–100%
Acute respiratory failure	[24,29,86]	22%–69%
Radiological Findings		
Bilateral interstitial infiltrates	[29,86]	89%–100%
Unilateral infiltrate	[29]	11%
Outcome		
ICU admission	[26,65,79]	8%–71%
Mechanical ventilation	[26,65,79]	8%–71%
Death	[24,26,29,65,79,86,94]	0%–27%
Death (total from all the studies)	[24,26,29,65,79,86,94]	25/173 (14%)

ICU: intensive-care unit.

### 4.2. Outcomes

In most studies, the outcome from PCP is generally described as being poorer in HIV-negative than in HIV-positive patients [21,22]. Hypoxemia has been reported to be more severe in HIV-negative than in HIV-positive patients. HIV-negative patients required higher oxygen-flow rates, more intensive-care admissions (50% *vs.* 35% for HIV-positive patients), and a greater need for invasive ventilation (30.5% *vs.* 11% for HIV-positive patients) [22]. Recent data concerning the outcomes of kidney-transplant recipients with PCP are presented in Table 5 and show there is high variability in the numbers of patients that are admitted into intensive-care units and need mechanical ventilation (8%–71%) [26,65,79].

PCP may also, in some cases, participate in graft loss, particularly in kidney-transplant recipients [71]. Hospital deaths are currently described as being higher in HIV-negative patients (27% *vs.* 4% for HIV-positive patients) [22]. Nevertheless, some reports have observed similar rates of mortality between these two populations, which may be explained by the increased use of adjunctive glucocorticoids in HIV-negative patients [18,51,115].

Mortality in the HIV-negative population varies according to the cause of the immunodeficiency [22]. Of interest, death rates seem to be lower for SOT recipients than for patients with other types of immunosuppression [22]. SOT has been identified as an independent variable associated with a lower death rate (OR 0.08, 95%CI: 0.02–0.31), and comparable to that of AIDS (OR 0.33, 95%CI: 0.12–0.92) [22].

The death rate among kidney-transplant recipients seems to have drastically decreased from about 50% before 1990 [47,116,117,118] to about 14% today (Table 5) [24,26,29,65,79,86,94]. Few recent data are available for other organ-transplant recipients, but death rates are about 7%–50% for liver- [25,38,94], 33% for lung- [25], and 5.5%–11% for heart-transplant [119,120] recipients.

A delayed diagnosis, resulting in a delay in the initiation of an adapted PCP therapy, can participate in a poor outcome, as the time from admission to initiation of PCP treatment is an independent predictor of mortality (OR 1.11/additional day, 95%CI: 1.04–1.18) [22]. Being older, needing oxygen or invasive mechanical ventilation on admission indicate a poor prognosis. A World Health Organization (WHO) performance status over 2, a lack of control of the underlying disease, a high temperature, hypoalbuminemia, shock, and clinical worsening by day 5 are also associated with increased mortality [121,122]. Pulmonary co-infections with herpes simplex virus or CMV and a high neutrophil count in a broncho-alveolar lavage (BAL) are also linked with more severe hypoxemia and higher mortality rates [123,124].

## 5. Microbiological and Radiological Diagnoses

### 5.1. Radiological Diagnosis

Radiographic imaging of HIV-negative patients is similar to that for HIV-positive patients with PCP. A typical radiographic feature of PCP is the presence of bilateral, symmetric, diffuse, reticular, or granular opacities [125], but these chest-radiographic images are non-specific and may be normal at diagnosis [126]. The interstitial infiltrates are mostly bilateral and are also frequently observed in SOT recipients (Table 5). These opacities are typically perihilar in mild presentations or diffuse in severe presentations. Typically, reticular and poorly defined ground-glass opacities progress to alveolar consolidation in 3–4 days [121].

High-resolution computed-tomography scans (HRCT) are more sensitive than chest radiography at detecting PCP [126]. HRCT can be indicated in HIV-negative patients with a normal chest radiograph [127]. HRCT may typically show ground-glass opacities, which predominate in the perihilar regions of lungs: however, these abnormalities are non-specific [128]. As their sensitivity and specificity are 100% and 89%, respectively, a normal HRCT may allow exclusion of PCP [129]. Thickened septal lines and areas of consolidation may be also present [130].

Pulmonary cysts occur in 3%–6% of non-HIV-patients with PCP and are associated with an increased risk of spontaneous pneumothorax [130]. Ground-glass opacities resolve completely after initiation of treatment in 97% of patients after a median period of 13 days [130]. Some differences in HRCT presentation exist according to HIV status, with a more rapid spread and a larger extent of ground-glass opacities, and a lower incidence of pulmonary cysts and a higher frequency of thickened septal lines described in HIV-negative patients than in AIDS patients with PCP [130].

### 5.2. Microbiological Diagnosis

There are important differences in the clinical presentation, outcomes and mortality of patients with PCP and with or without AIDS. These differences also concern the diagnosis in HIV-negative patients with PCP, including SOT recipients, who have a lower burden of *P. jirovecii* than those with AIDS, which leads to difficulty in detecting the fungus [131].

#### 5.2.1. Samples

Microbiological diagnosis of PCP is possible in a great variety of pulmonary specimens (Table 6). However, the most appropriate sample has to be selected by taking into account the best diagnostic sensitivity and the lower invasiveness of the sampling procedure. Due to the importance of minimal invasiveness and the development of sensitive methods to make a diagnosis, such as real-time PCR, transbronchial or an open-lung biopsy, are now rarely used in most centers and a diagnosis of PCP is preferentially obtained from a BAL fluid sample or induced sputum. Although BAL fluid remains the sample of choice, induced sputum is a rapid and cost-effective diagnostic procedure, and is less invasive than bronchoscopy [52,121]. At this time, the development of highly sensitive molecular diagnosis tools has allowed less invasive sampling of the upper respiratory tract, such as oral washing or aspiration [132,133]. New approaches, such as detection of the β-d-glucan antigen in blood samples, also helps facilitate in the diagnostic management of PCP [134].

**Table 6:** Numbers of *Pneumocystis* pneumonia (PCP) diagnosed with traditional diagnostic methods according to the type of samples. Adapted from Rodriguez *et al.* [31].

Type of Sample	Reference	PCP Diagnosed (%)
Routine sputum	[114,135]	Poor
Induced sputum	[136,137]	30–55
Bronchoalveolar lavage	[137,138]	80–95
Bronchoalveolar lavage and transbronchial biopsy	[137,139]	>95
Open-lung biopsy	[140]	>95

#### 5.2.2. Traditional Diagnostic Methods

As *Pneumocystis* remains a non-cultivable microorganism, traditional diagnostic methods rely on microscopic observations of the pathogen in respiratory specimens. During infection, two life-cycle forms can be found: the asci (formerly named cysts) and the trophic form, with the latter being more dominant than the asci form in PCP [141]. Trophic forms can be detected with modified Papanicolaou, May-Grünwald Giemsa, Giemsa, or Gram-Weigert stains. *P. jirovecii* asci can be stained with Gomori methenamine silver, toluidine blue, cresyl echt violet, or calcofluor white [142]. An immunofluorescence assay with anti-*Pneumocystis* antibodies is commercially available: it provides the best specificity and sensitivity of the general stains [136] and some immunofluorescent monoclonal antibodies can reveal both trophic and asci forms. Before the development of PCR, this method represented the “gold standard” technique for the diagnosis of PCP.

Although microscopic methods are often sufficient to diagnose *P. jirovecii* in BAL fluids from AIDS patients, the sensitivity of these methods is often too low to diagnose PCP in non-HIV immunocompromised patients [124]. In SOT recipients, molecular diagnostic methods are required to avoid missing a *Pneumocystis* infection in this population.

#### 5.2.3. Diagnoses with the Polymerase Chain Reaction

The use of the polymerase chain reaction (PCR) assays has improved the sensitivity of detecting *Pneumocystis*, particularly in non-HIV-patients. Numerous assays using various gene targets and different PCR-detection methods (single-step, nested, semi-nested, real-time PCR) have revealed PCR to be highly effective at diagnosing PCP in both HIV-positive and HIV-negative patients [77,143,144,145,146,147]. The high negative predictive value of this method, close to 100%, allows PCP to be excluded with a high probability and cotrimoxazole therapy can then be discontinued when PCR is negative [148].

Among the numerous gene targets evaluated in different studies (dihydropteroate synthase, dihydrofolate reductase, internal transcribed spacer regions of the rRNA gene, mitochondrial large subunit ribosomal RNA, 5S rRNA, b-tubulin, major surface glycoproteins, kex-1, cdc2 gene loci), sensitivity seems to be generally highest in assays directed at multicopy targets, such as the major surface glycoproteins (MSG) and the mitochondrial large subunit ribosomal RNA (mtLSU rRNA) genes [77].

Conventional PCR (nested or hemi-nested) has a higher sensitivity than traditional diagnostic methods, but suffers from several limitations. This test has low specificity, high false-positive rates, and low positive predictive values, and cannot differentiate PCP from asymptomatic *Pneumocystis* colonization. Moreover, the possibility of run-to-run contamination is high, limiting its utility in diagnostic practice [144,146,149]. Real-time quantitative PCR (qPCR) assays have been developed to improve test specificity and semiquantitative results provided by these methods may help differentiation between PCP and *Pneumocystis* colonization [146,149].

Reported performances for PCP diagnoses have been excellent, particularly in assays directed at MSG and mtLSU rRNA genes (Table 7), with sensitivities from 93% to 100% and specificities higher than those observed for standard PCR (83%–100%). Other advantages of this method are the reduced turnaround times (<3 h) and the reduced possibility of run-to-run contamination [77]. Clinically relevant cut-off PCR values of fungal load have been proposed to discriminate between colonization and PCP entities. Nevertheless, as most studies concern non-commercial PCR kits, the extrapolation of a threshold from one technique to another is difficult [121]. In several studies, the number of DNA copies per tube or the number of trophic-form equivalents have been proposed to discriminate active pneumonia from colonization [150,151,152]. In other studies, cycle thresholds (*C*t) of the qPCR assay have been used as a “cut-off” to distinguish between the two clinical entities [153,154]. As significant fluctuations in the amount of fungal DNA may occur, because of the variable amounts of saline used in the lavage procedure and/or the pre-analytic factors such as sample transport conditions [77], thresholds based on *C*t seem to have sufficient precision to guide towards colonization or PCP. Nevertheless, clinical judgment and radiological presentation are essential to distinguish PCP from a simple colonization, and more so if the fungal burden is low.

**Table 7:** Major studies that have evaluated a diagnosis of *Pneumocystis* pneumonia (PCP) using real-time PCR directed at major surface glycoproteins (MSG) and mitochondrial large subunit (mtLSU) rRNA genes, in non-HIV immunocompromised patients. Adapted from Reid *et al.* [77].

Study	Target Gene	Specimen (No.)	No. PCP Episodes	Sensitivity (%)	Specificity (%)	PPV (%)	NPV (%)
Flori *et al.* [146]	mtLSU rRNA	BAL (173)	11	100	87	NA	NA
Dini *et al.* [155]	mtLSU rRNA	Respiratory tract (932)	150	100	83 ^a^	78.1 ^a^	100 ^a^
Hauser *et al.* [156]	mtLSU rRNA	Respiratory tract (110) BAL (101)	14	93	90–91	59–65	98–99
Alanio *et al.* [152]	mtLSU rRNA	BAL (163) IS (115)	16	100	85.7	72.4 ^a^	100
Flori *et al.* [146]	MSG	BAL (173)	11	100	98.6	84.6 ^a^	100 ^a^
Alvarez-Martinez *et al.* [157]	MSG	BAL, IS (213)	111	100	90.2 ^a^	82 ^a^	100 ^a^
Fillaux *et al.* [153]	MSG	BAL (400)	66	100	90.5	47	100
Chumpitazi *et al.* [151]	MSG	BAL (66)	18	100	97.7	95.5	100

BAL: bronchoalveolar lavage; IS: induced sputum; NA: not available; NPV: negative predictive value; PPV: positive predictive value. ^a^ Calculated from data presented in a respective publication and in Chumpitazi *et al.* [151].

Moreover, as a period of colonization most likely precedes the development of acute PCP [158], this colonization should not be overlooked. Furthermore, “colonization” may have several important clinical implications because of stimulation of the inflammatory response within the lung [159,160,161]. As shown in chronic obstructive pulmonary disease, colonization may be involved in the development or progression of various lung diseases, suggesting the possible utility of treating these colonizations [162]. Finally, ongoing arguments suggest that a strict dichotomization of PCP and colonization is artificial because of a probable continuum of Pneumocystis infections from severe hypoxemia PCP to transient colonization in the immunocompetent.

## 6. Plasmatic Markers

As a non-specific marker for lung injury, the level of serum lactic dehydrogenase is elevated (>300 IU) in most patients with PCP [31]. Lactic dehydrogenase level can help indicate PCP [24] and correlates with severity of the disease [16]. However, this marker is not specific and is also increased in other pulmonary diseases [31].

There has been recent interest in using serum levels of β-d-glucan (BDG), a polysaccharide present in the *Pneumocystis* cyst wall but also in most fungi, as a noninvasive diagnostic test for PCP. Performance of commercial BDG assays have been examined in several studies with sensitivities ranging from 90% to 100% and specificities of 88%–96% in non-HIV-infected immunosuppressed patients with pneumonia [134,162,163,164]. However, the standardized cut-off values for clinical infection have not been determined. Strategies to distinguish colonization from PCP are also proposed using BDG levels [165,166]. Although one study found a relationship with organism burden, BDG levels do not correlate with disease severity or the response to therapy, which makes this marker unsuitable for monitoring a response to treatment [134,164,167]. Moreover, BDG is present in the walls of most fungi [168,169] and its level is also increased in other fungal infections. Bacterial pneumonia [170] or β-lactam antibiotic administration (particularly piperacillin-tazobactam) [171] may also lead to false positive results. For this reason, BDG can be useful as an ancillary test in patients with a high suspicion of PCP.

## 7. Prophylaxis

### 7.1. General Considerations

While guidelines for PCP prophylaxis among HIV patients [172] are specific and universally accepted, there is a lack of consensus on the prophylaxis for other immunocompromised people. In SOT recipients, the highest risk period for the development of PCP is the first six months after transplantation [33,45,46,47,48,49,50], with a PCP incidence of about 10% (5%–15%) [16,28,29]. The use of a post-transplantation primary prophylaxis is now commonly accepted by the majority of the experts: nevertheless, the duration of treatment is still debated. In 2002, the European Renal Association recommended a PCP prophylaxis period of at least four months post-transplantation [173]. More recently, the Kidney Disease Improving Global Outcomes guidelines recommended 3–6 months [174], and the American Society of Transplantation Guidelines recommended 6–12 months [69,111] after transplantation [174] for all recipients, although longer durations could be considered [111]. Indeed, some data suggest that treatment duration can be prolonged to >1 year [25,31].

Although lifelong prophylaxis is not recommended for kidney-transplant recipients, it may be indicated for lung-, heart- and small-bowel-transplant recipients as the incidence of PCP is higher in this population [31,111]. Even if no consensus exists on the optimal duration of primary PCP prophylaxis, its generalized use in SOT recipients during the first months post-transplantation has drastically decreased the incidence of this infection during this period [16,175].

However, in many cases, PCP has occurred after the recommended post-transplantation prophylaxis period [27,176]. After this period, there are no clear recommendations for the re-initiation of anti-PCP prophylaxis. The risk threshold estimated by a peripheral CD4+ T-cell count of <200/μL and commonly used for HIV-positive patients [101,177] seems to not apply to HIV-negative immunocompromised patients and particularly not to SOT patients [20,122,178]. Instead, PCP risk factors identified in SOT recipients may help guide indications for chemoprophylaxis.

Patients with a history of a prior PCP infection, cytomegalovirus (CMV) infection, lymphopenia, prolonged neutropenia, or who require higher than usual levels of immune suppression, [31,32,111] may benefit from PCP prophylaxis, even if no clear consensus of duration is available. Strategies to target patients at high risk for PCP after the first year post-transplantation, in SOT recipients who benefit from initial TMP-SMX prophylaxis [27], using their age, date of transplantation, lymphocyte count, and presence of detectable CMV viremia, may also be considered. In addition, PCP prophylaxis is recommended for kidney-transplant recipients for at least six weeks during and after treatment for an acute rejection [174].

### 7.2. Trimethoprim-Sulfamethoxazole (TMP-SMX)

TMP-SMX remains the drug of choice to prevent PCP in HIV-positive [179] or -negative patients [32,111] (Table 8). Several studies have shown the effectiveness of TMP-SMX prophylaxis in SOT recipients [30,41,49,175,180]. A Cochrane review showed that TMP-SMX prophylaxis was highly effective at preventing PCP in both HIV-negative children and adults [181]. Overall, TMP-SMX shows an 85% reduction in the incidence of PCP infections (RR 0.15, 95%CI: 0.04–0.62) and PCP-related mortality was reduced by 83% (RR 0.17, 95%CI: 0.03–0.94) [181]. For SOT recipients the RR was 0.09 (95%CI: 0.02–0.48) [181]. The common adult dose of TMP-SMX was 160/800 mg and it can be administered thrice weekly, as its efficacy is similar to once daily administration. The overall prevalence of severe adverse events with TMP-SMX is low. In the Cochrane review, no severe adverse events were observed with TMP-SMX among the trials that compared TMP-SMX to a placebo or no treatment [181]. Overall, in all the studies included in this meta-analysis that focused on TMP-SMX treatment, severe adverse events that required permanent discontinuation, including leukopenia, thrombocytopenia, or severe dermatological reactions, occurred in 3.1% of adults and 0% of children [181]. The most serious adverse effect was leukopenia [108,181]. Prophylactic TMP-SMX should be considered when the risk of PCP is >6.2% overall in patients not receiving prophylaxis [181], which includes for the first six months for those who have undergone solid-organ transplantation [31]. During outbreaks of PCP among immunocompromised patients, the incidence of PCP may be high enough to justify prophylaxis.

**Table 8:** Mean prophylactic and therapeutic options for *Pneumocystis* pneumonia (PCP). Adapted from Martin *et al.* [111] and Catherinot *et al.* [52].

Drug	Prophylactic Regimen	Curative Regimen	Comments
Trimethoprim—sulfamethoxazole	TMP: 80–160 mg—SMX: 400–800 mg, daily *p.o.* or IV TMP: 160 mg—SMX: 800 mg, 3 times weekly *p.o.* or IV	TMP: 15–20 mg/kg—SMX: 75–100 mg/kg, IV or *p.o.*, divided into 3–4 doses daily	Contraindication in cases of allergy to sulfa drugs. Adverse effects: Cytopenia, skin reactions, hepatitis, pancreatitis, gastrointestinal disturbance, renal insufficiency, hyperkalemia, anaphylaxis. Interaction with cyclosporine: increase of creatinine level with possible decrease of cyclosporine plasmatic concentrations.
Dapsone	50–100 mg, daily *p.o.*	-	Contraindication in cases of G6PD deficiency. Possible cross-reaction with sulfa allergy. Adverse effects: methemoglobinemia, anemia, skin rash, gastrointestinal disturbance, agranulocytosis
Atovaquone	750 mg, 2 times daily *p.o.*	750 mg, 2–3 times daily *p.o.*	Variable oral absorption. Adverse effects: skin rash, fever, gastrointestinal disturbance, hepatitis
Pentamidine	300 mg, monthly administered through aerosolized nebulizer	4 mg/kg, daily IV	Adverse effects: pancreatitis, hypo- or hyperglycemia, bone-marrow suppression, renal insufficiency, cardiac arrhythmias, electrolyte disorders, hypotension, hepatitis. Interaction with others nephrotoxic drugs, increasing renal toxicity, particularly cyclosporine or tacrolimus
Dapsone + trimethoprime	-	Dapsone: 100 mg, daily *p.o.* Trimethoprime: 5 mg/kg, 3 times daily	Contraindication in cases of G6PD deficiency. Possible cross-reaction with sulfa allergy. Adverse effects : methemoglobinemia, skin rash, fever, gastrointestinal disturbance
Clindamycin + primaquine	-	Clindamycin: 600 mg, 4 times daily IV or 350–400 mg, 4 times daily *p.o.* Primaquine: 15–30 mg daily *p.o.*	Contraindication in cases of G6PD deficiency. Adverse effects: skin rash, fever, neutropenia, gastrointestinal disturbance, methemoglobinemia. Interaction with cyclosporine: possible decrease of cyclosporine plasmatic concentrations.

IV: intravenous. *p.o.*: *per os.*

### 7.3. Other Chemoprophylactic Agents

#### 7.3.1. Dapsone

Dapsone is considered a second-line agent to prevent PCP after TMP-SMX therapy [111,182] (Table 8). It can be used in combination with pyrimethamine or trimethoprim. Because of its long half-life in plasma, dapsone may be administered in dosages of 50–100 mg daily to 100 mg weekly. However, dapsone must be avoided in G6PD or methemoglobin-reductase deficiency. Adverse events include agranulocytosis, aplastic anemia, rash, nausea, and sulfa reactions [183]. Intolerance may be more common among solid-organ transplant recipients [184] and often in those who are intolerant of TMP-SMX. A switch from TMP-SMX to dapsone is generally not recommended for patients with desquamation, neutropenia, interstitial nephritis, or hepatitis.

#### 7.3.2. Atovaquone

Atovaquone is an inhibitor of the mitochondrial electron transport. In HIV patients, atovaquone seems to be equivalent to dapsone in preventing PCP [185] (Table 8). In solid-organ transplant recipients it appears to be effective and well tolerated [32,186] and may be an alternative PCP prophylaxis despite its unknown optimal dosage and its extremely variable absorption. Nevertheless, failures of atovaquone have been reported in patients taking 1000 mg or less daily [31,32]. The most common side effects generally do not require treatment to be discontinued: *i.e.*, rash, headache, nausea, and diarrhea [35,187].

#### 7.3.3. Pentamidine

Inhaled pentamidine (300 mg, every 3–4 weeks), administrated as droplets from a nebulizer, should be considered as a third-line agent [111] (Table 8). Pentamidine is well-tolerated with minimal side effects other than bronchospasms or a cough. Toxicity has been observed in 3%–7.5% of SOT recipients [188,189]. Nevertheless, it seems to be less effective than TMP-SMX, dapsone, or atovaquone, as the use of inhaled pentamidine has been associated with a higher incidence of breakthrough infections. Disseminated *Pneumocystis* infection [190,191] or PCP predominating in the upper lung lobes, with cystic formation and pneumothorax development [192,193] has occurred in HIV-patients receiving aerosolized pentamidine.

#### 7.3.4. Other Agents or Combinations

Other agents or combinations (sulfadoxine-pyrimethamine, TMP-dapsone, clindamycin-primaquine, intravenous pentamidine) have been used with variable results but data are often insufficient to recommend these agents in practice [31].

### 7.4. Prophylaxis and Nosocomial Infections

As PCP outbreaks have been largely documented among kidney-transplant recipients [60,61,62,63,66,106,107], some authors recommend isolation of immunocompromised patients with PCP or patients in whom pulmonary colonization with *P. jirovecii* is diagnosed [194]. The use of a facemask to prevent aerial transmission of *Pneumocystis* may also be recommended to protect susceptible patients [63].

## 8. Treatments

### 8.1. Anti-Pneumocystis Agents

#### 8.1.1. Trimethoprim-Sulfamethoxazole (TMP-SMX)

TMP-SMX is the first-line agent for the treatment of mild to severe PCP in both HIV and non-HIV-infected patients [195] (Table 8). Its combination with other anti-*Pneumocystis* agents is not recommended in the first instance [121]. TMP-SMX acts by interfering with folate metabolism (dihydrofolate reductase and dihydropteroate synthase) in *Pneumocystis*. TMP-SMX is also the drug of choice for SOT patients [111] because of its high efficacy and its availability in oral and parenteral forms. The recommended daily dose is TMP 15–20 mg/kg plus SMX 75–100 mg/kg, preferably by IV administration for severe PCP.

Monitoring of SMX drug levels is controversial for patients receiving IV therapy and no practical information is available in the current guidelines [196]. As TMP-SMX has excellent oral bioavailability, with concentration in serum most of the time, comparable to intravenous (IV) administration, oral administration seems adequate for non-severe patients who are able to take *per os* medications. Adverse reactions usually begin during the second week of treatment, but they occur less commonly in HIV-negative patients than in HIV-positive patients [110]. Recommended duration of treatment is 21 days in HIV-positive patients because of their higher fungal load and slower clinical response, with a higher risk of relapse after treatment [197]. For HIV-negative patients, such as SOT recipients, the optimal duration has not been fully studied but is generally 14 days [121]. This duration of therapy can be extended to 21 days in severe infections or when clinical improvement is prolonged [111].

The effectiveness of anti-*Pneumocystis* treatment (when corticosteroids are not associated) cannot be evaluated in the first days after beginning therapy as patients usually worsen during this period and only start to improve after 4–8 days [110]. If clinical improvement is not observed after 4–8 days, changes to the treatment should be made with an alternative IV drug. If the first-line agent was oral TMP-SMX, a switch can be made to an IV regimen [121]. Mutations in dihydropteroate synthetase (DHPS) have been described but no clear association between these mutations and failure of TMP-SMX treatment or altered outcomes have been demonstrated in AIDS patients [198,199].

#### 8.1.2. Other Agents

Intravenous pentamidine is about as effective as TMP-SMX in AIDS-patients [200,201,202] and remains probably the best second-line agent after TMP-SMX for SOT recipients [111] (Table 8). Nevertheless, its use can be complicated by numerous toxicities in 71% of patients, leading to drug withdrawal in 18% [203]. Due to the potential for islet-cell necrosis, pentamidine must be avoided in cases of pancreas or islet transplantation [111]. Atovaquone, clindamycin-primaquine, or dapsone-TMP have only been evaluated in mild to moderate PCP in AIDS patients (Table 8). The clindamycin-primaquine combination seems to be the most effective regimen, particularly in cases where TMP-SMX has failed, as is associated with better outcomes as a second-line treatment compared to pentamidine in AIDS patients [204].

Few clinical data support the use of pyrimethamine–sulfadiazine, macrolide-SMX, or caspofungin-TMP-SMX, and the data are insufficient to recommend these agents in practice [205,206].

### 8.2. Adjuvant Corticosteroids

Adjunctive glucocorticoids are recommended for HIV-positive patients with moderate to severe PCP [197], defined as PaO_2_ <70 mmHg while breathing ambient room air. The benefit to survival from corticosteroids begins during the first 72 h of treatment [52]. In HIV-negative patients with moderate-to-severe PCP, the use of adjunctive glucocorticoids remains questionable and is highly controversial [207,208]. No prospective trial has been conducted on HIV-negative patients. Recommendations are based on small-scale retrospective analyses, including very heterogeneous populations of patients, varying dosages of corticosteroids, and the conclusions are contradictory.

In four retrospective studies, there was no significant improvement in outcome with adjunctive steroid therapy [113,123,209,210], although Bolée *et al.*’s study [113] found a trend for longer survival in patients who received adjunctive steroids (*p* = 0.07). In Pareja *et al.*’s study, there was no difference in the mortality rates of patients treated with adjunctive high-dose steroids but they did spend less time on mechanical ventilation compared to patients not managed with steroids [18].

KDIGO and the American Society of Transplantation guidelines recommend treatment with adjunctive corticosteroids for SOT recipients with moderate to severe PCP (as defined by PaO_2_ <70 mmHg in room air) [111,174]. Corticosteroids should be administered with antimicrobial therapies, ideally within 72 h of initiating the antimicrobial therapy to obtain the maximum benefits [111]. The optimal dose of corticosteroids has not been well-established. American Society of Transplantation guidelines suggest that 40–60 mg of prednisone is administered *per os* twice daily and tapered after 5–7 days over a period of 1–2 weeks [111]. Nevertheless, in a recent retrospective study that included 139 HIV-negative patients with PCP, Lemiale *et al.* suggested that adjunctive high doses of corticosteroids (>1 mg/kg/day) increased the death rate [211]. Even though this study suffers from several limitations, such as having a retrospective design, a 23-year period of inclusion, a lack of information on the factors that governed the use or timing of corticosteroid initiation, these data challenge the recommendations on the use of corticosteroids for SOT recipients with PCP.

### 8.3. Reduction in Immunosuppressive Medications

The National Kidney Foundation recommends reducing immunosuppressive medications for kidney-transplant recipients who develop PCP [174]. Thus, this recommendation may logically be enlarged to all SOT recipients with PCP.

## 9. Conclusions

PCP remains an important cause of morbidity and mortality in SOT patients, even though the early post-transplantation period now carries a much lesser risk of infection because of the use of primary TMP-SMX as a prophylaxis. However, the occurrence of PCP after completion of a course of prophylaxis has now become problematic, particularly during the second year post-transplantation. As systematic PCP prophylaxis after the first year does not seem to be appropriate because of the low overall proportion of SOT patients who develop PCP, an individualized approach to target patients at risk seems to be a more relevant strategy. Because of the lack of biological parameter associated with a high risk for PCP, as occurs for AIDS patients, criteria based on risk factors identified for these patients, such as CMV infection, graft rejection, or lymphopenia, could be helpful in this context.

Even though a recent study shows that mortality seems to be lower in SOT recipients than in other types of immunocompromised patients, and comparable to that of AIDS patients, the prognosis remains closely related to the precocity of diagnosis and treatment. In SOT recipients, microbiological diagnosis must not be based on traditional microscopic methods. Molecular methods are highly effective in the diagnosis of PCP and have a highly negative predictive value close to 100%, which allows exclusion of PCP in SOT recipients with pneumonia. PCR methods or new approaches, such as detection of the β-d-glucan antigen, also facilitate a diagnosis of PCP in patients who cannot benefit from invasive sampling.

TMP-SMX is consensually considered as the first-line anti*-Pneumocystis* agent, but the use of adjunctive glucocorticoids remains highly controversial and recommendations are based on small-scale retrospective studies as no prospective trial has been conducted on SOT recipients. In contrast to AIDS-positive patients, the most recent data suggest that a high dose of adjunctive glucocorticoids may be deleterious in HIV-negative patients.
